# Plausible Involvement of Ethylene in Plant Ferroptosis: Prospects and Leads

**DOI:** 10.3389/fpls.2021.680709

**Published:** 2021-06-28

**Authors:** Riyazuddin Riyazuddin, Ravi Gupta

**Affiliations:** ^1^Department of Plant Biology, Faculty of Science and Informatics, University of Szeged, Szeged, Hungary; ^2^Doctoral School in Biology, Faculty of Science and Informatics, University of Szeged, Szeged, Hungary; ^3^Department of Botany, School of Chemical and Life Sciences, Jamia Hamdard, New Delhi, India

**Keywords:** ferroptosis, reactive oxygen species, glutathion (GSH), nicotinamide adenine dinucleotide phosphate oxidase, ethylene

## Introduction

Plants are continuously exposed to various biotic and abiotic stressors that limit their productivity. Any kind of stressor leads to the generation of Reactive Oxygen Species (ROS), majorly, through the activity of plasma membrane-localized respiratory burst oxidase homolog (RBOH) proteins that generate O_2_^•-^ in the apoplast (Mittler, 2017). Owing to its reducing properties, O_2_^•-^ is highly toxic and is involved in the peroxidation of membrane lipids and the conversion of Fe^3+^ to ferrous ions (Fe^2+^). In addition, O_2_^•-^ can also be converted into H_2_O_2_ by the activity of superoxide dismutase (SOD), which may enter the cytosol through aquaporins to trigger signaling cascades (Rodrigues et al., 2017). Further, Fe^2+^ can directly interact with H_2_O_2_ through Fenton reaction and accelerates the production of hydroxyl radical (OH•), which is another lethal ROS (Mittler, 2017). Since ions of Fe play a major role in ROS production, plants keep a check on the concentration of these ions to maintain ROS homeostasis. In plants, two major groups of proteins participate in iron (Fe^2+^/Fe^3+^) sequestration including ferritin and plant defensin proteins. The latter group of proteins also participate in the plant defense and inhibit the growth of fungal pathogens. Recently, a pivotal role of iron in the hypersensitive response induced cell death, during plant defense signaling, was reported in rice upon infection with the fungal pathogen, *Magnaporthe oryzae* (Dangol et al., 2019). This type of iron-dependent cell death was first reported in mammalian tumor cells and was termed “ferroptosis” by Dixon and co-workers in 2012 (Dixon et al., 2012). Ferroptosis is, genetically, morphologically, and biochemically, distinct from other types of known cell death including apoptosis, necrosis, and autophagy (Dixon et al., 2012). The onset of ferroptosis requires high levels of ROS and Fe^2+^ and depletion of reduced glutathione (GSH) (Dixon and Stockwell, 2019). Subsequently, a GSH-dependent glutathione peroxidase-4 (GPX-4) was identified as a key player of mammalian ferroptosis (Angeli et al., 2014; Yang et al., 2014). GPX enzymes catalyze the reduction of H_2_O_2_ or organic hydroperoxides to water or corresponding alcohols using reduced GSH or thioredoxin (TRX) (Yang et al., 2016).

In plants, ferroptosis was first reported by Distéfano and co-workers in 2017 in *Arabidopsis thaliana* under heat stress conditions, and, similar to the animals, the plant ferroptosis was also found to be dependent on the GSH depletion, lipid peroxidation, and accumulation of ROS and ions of Fe (Distéfano et al., 2017). Although the literature evidence on plant ferroptosis is limited, it has been observed that it can be triggered by both abiotic (heat stress) and biotic stressors (*M. oryzae)*, and both plant and animal ferroptosis exhibit similar morphological characteristics, such as cytoplasmic retraction, normal nuclei, and the formation of small lytic vacuoles (Distéfano et al., 2021). In addition, a noticeable shrinkage of mitochondria along with increased mitochondrial membrane density and decreased mitochondrial cristae has been observed in animal ferroptosis (Dixon et al., 2012); however, no such observation has been reported in the case of plants. By utilizing several agonists (Supplementary Table 1), Dangol et al. characterized the detailed process of plant ferroptosis, and their results collectively suggested the role of plasma membrane-localized NADPH-oxidases (RBOHs), NADP-malic enzyme, polymerization of actin microfilaments along with the depletion of GSH, peroxidation of membrane lipids, and accumulation of ions of Fe and ROS (Dangol et al., 2019). Similar to the animals, the plant ferroptosis also seems to be dependent on the activity of GPX-4 as treatment for erastin, an inducer of ferroptosis and inhibitor of GPX-4, resulted in the depletion of reduced and total GSH and accumulation of ROS and Fe^3+^ to result in ferroptosis in rice sheath cells (Dangol et al., 2019). Correspondingly, treatment of Arabidopsis cells with another GPX-4 inhibitor RSL3 led to cell death, which was prevented by the treatment of ferroptosis inhibitors including liproxstatin-1 and ferrostatin-1 (Hajdinák et al., 2019). A recent study demonstrated that silencing of *GPX4* in *Nicotiana benthamiana* resulted in enhanced ferroptosis in response to *Tobacco mosaic virus* 24A+UPD infection (Macharia et al., 2020). However, some of the recent evidence suggests the possible involvement of others GPXs, such as membrane-localized GPXL5, in the plant ferroptosis (Meyer et al., 2020). Overall, these results suggest that ferroptosis is a highly regulated cell death process that is induced by both biotic and abiotic stressors. Since the analysis of ferroptosis during incompatible rice–*M. oryzae* interactions suggested a positive role of ferroptosis in preventing the infection by avirulent strains of the fungus, the process of ferroptosis can be manipulated in the future to develop the biotic and abiotic resilient crops (Dangol et al., 2019; Kazan and Kalaipandian, 2019).

### Evidence in Support of Ethylene Mediated Regulation of Ferroptosis

Although the direct involvement of any phytohormones in the plant ferroptosis has not been reported yet, a growing body of evidence suggests the possible involvement of ethylene in the plant ferroptosis. It has been observed that all the components of ferroptosis, including GSH, ions of Fe, and ROS, are linked to the gaseous plant hormone ethylene, and thus, we hypothesize the involvement of ethylene in the regulation of plant ferroptosis based on the following literature evidence.

1. There is ample evidence that suggests ethylene induces the biosynthesis of GSH. For instance, exogenous treatment of ethylene has been shown to significantly elevate the levels of GSH and ascorbate (AsA) in *Zea mays* seedlings under cadmium (Cd) toxicity (Liu et al., 2019). In addition, the exogenous application of ethephon, an ethylene precursor, increased the amount of GSH in Cd-treated *Brassica juncea* (Khan et al., 2016), while the application of ethylene inhibitor Aminoethoxyvinylglycine (AVG) resulted in decreased GSH levels (Masood et al., 2012). Similarly, ethephon treated *Glycine max* plants showed an accumulation of GSH together with the increased activity of glutathione reductase (GR) under waterlogging conditions (Kim et al., 2018). In contrast, treatment using ethylene biosynthesis inhibitor resulted in reduced levels of GSH in Cd-exposed *Lycium chinense* plants compared with untreated plants, further confirming a direct role of ethylene in the regulation of cellular GSH levels (Guan et al., 2015).
2. The connection between Fe^2+^ and ethylene is well-established, and it has been known for years that excess ions of Fe trigger ethylene biosynthesis in plants (Peng and Yamauchi, 1993; Becker and Asch, 2005; Majerus et al., 2007). The ethylene biosynthetic pathway is relatively simple, taking place *via* only two committed enzymatic reactions catalyzed by 1-amino-cyclopropane-1-carboxylic acid (ACC) synthase (ACS) and ACC oxidase (ACO) (Riyazuddin et al., 2020). Both ACS and ACO are controlled at the transcriptional and post-translational levels, which enables a tailored regulation of ethylene production in plants (Pattyn et al., 2021). The final regulatory step of the ethylene biosynthetic pathway is the conversion of ACC to ethylene and is catalyzed by ACO, which requires Fe^2+^ as the active-site cofactor. Therefore, Fe^2+^ plays a critical role in ethylene biosynthesis by regulating the activity of ACO (Houben and Van de Poel, 2019). Subsequently, the deficiency or accumulation of Fe^2+^ may directly affect cellular ethylene production. In other words, Fe^2+^ may be the limiting factor in ethylene production, and the excess could result in higher production of ethylene (Peng and Yamauchi, 1993; Li et al., 2015). For instance, excess Fe^2+^ resulted in the upregulation of the transcript level of ethylene biosynthesis genes, such as *AtACS2, AtACS7, AtACS8, AtACS11*, and *AtACO1* and *AtACO2*, and contributed to higher ethylene production in Arabidopsis (Li et al., 2015). In turn, ethylene, thus, produced triggered the expression of genes encoding Fe-sequestering ferritins, such as *FER1, FER2, FER3*, and *FER4*, and minimized Stelar and xylem Fe^2+^ concentrations to limit Fe accumulation and toxicity both in shoots and roots (Li et al., 2015). Further, *FER1* and *FER3* were found to be significantly elevated in the roots of Arabidopsis ethylene-overproduction mutant (*eto1-1*) as compared with wild type (WT) during Fe^2+^ toxicity (Li et al., 2015).
3. The interconnection between ethylene and ROS homeostasis is quite evident, and it is well-known that ethylene maintains ROS homeostasis by activating the enzymatic and non-enzymatic antioxidant defense to limit the accumulation of ROS and subsequent peroxidation of membrane lipids that result in ferroptosis (Riyazuddin et al., 2020). At first, it was shown that the exogenous application of ethylene precursor ACC resulted in the enhanced activities of ascorbate peroxidase (APX), catalase (CAT), SOD, and peroxidase (POX), and reduced the lipid peroxidation in creeping bentgrass (Larkindale and Huang, 2004). Similarly, exogenous application of ACC has been shown to improve the heat stress tolerance in rice seedlings by reducing lipid peroxidation and relative electrolyte leakage during heat stress (Wu and Yang, 2019). Correspondingly, the exogenous application of ethephon, another ethylene precursor, to Ni-treated *Brassica juncea* plants resulted in significantly increased activity of SOD, APX, GR, GPX, and the accumulation of proline (Khan et al., 2020). Similarly, exogenous application of ethephon substantially induced the activity of enzymatic and non-enzymatic antioxidants, such as SOD, APX, GR, GSH -S-transferase (GST), GPX, monodehydroascorbate reductase (MDHAR), dehydroascorbate reductase (DHAR), AsA, and GSH, under Zinc toxicity in *B. juncea* (Khan et al., 2019). In *Lactuca sativa*, the application of exogenous ethylene increased the activity of antioxidant enzymes, such as SOD, CAT, and APX, and reduced the H_2_O_2_ content (Ma et al., 2013), while in *Nelumbo* sp., ethylene treatment maintained endogenous ROS levels by regulating the AsA–GSH antioxidant system under Cd stress (Yuan et al., 2018). In *Dendrobium nobile*, ethephon treatment led to an increase in the contents of AsA and GSH, while exogenous treatment of ethylene inhibitor resulted in decreased contents of both of these antioxidants. In addition, APX and GR also showed a significant change under the ethylene regulator treatments (Zhang et al., 2021). Further, a growing body of evidence suggests that Ethylene response factors (ERFs) play a role in linking redox and hormonal regulation in plant responses to abiotic stresses. The overexpression of *ERF96* gene enhanced selenium tolerance in Arabidopsis *via* elevation of CAT, GPX activities, and GSH content to cope with H_2_O_2_ compared with those in WT (Jiang et al., 2020). Similarly, overexpression of *ERF38* resulted in reduced contents of MDA and H_2_O_2_ in the transgenic poplars as compared with WT by elevating the expression of POD and SOD and accumulation of proline and soluble proteins (Cheng et al., 2019). Furthermore, overexpression of *ERF1* in tomatoes resulted in higher proline accumulation and lower lipid peroxidation as well as increased the activity of antioxidant enzymes (POD and SOD) under salt stress (Hu et al., 2014). ERF3 also regulated ROS metabolism in tobacco resulting in lower accumulation of ROS (Wu et al., 2008). Overall, all of these results confirm a tight correlation between ethylene levels and ROS homeostasis in plants.
4. In addition to regulating the enzymatic and non-enzymatic antioxidants, ethylene has also been shown to regulate the activities of NADPH-oxidases (RBOHs) to limit ROS production. At first, ethylene was shown to be involved in the regulation of H_2_O_2_ signaling by controlling the expression of *Rboh* genes under hypoxia stress in Arabidopsis (Yang and Hong, 2015). In addition, ACS1 mediated early ethylene production has been shown to temporarily inhibit the expression of NADPH-oxidase (RBOH-D and RBOH-F) genes to prevent the ROS burst in *Brassica oleracea* (Jakubowicz et al., 2010). On the contrary, reduced expressions of ethylene biosynthesis genes including *ACS7* and *ACS8* and ethylene signaling genes including *ERF73* were observed in the *rbohd*-knockout mutants, further indicating an intricate relationship between NADPH-oxidase, ROS production, and ethylene (Yang and Hong, 2015).

## Conclusion

**Figure 1:**
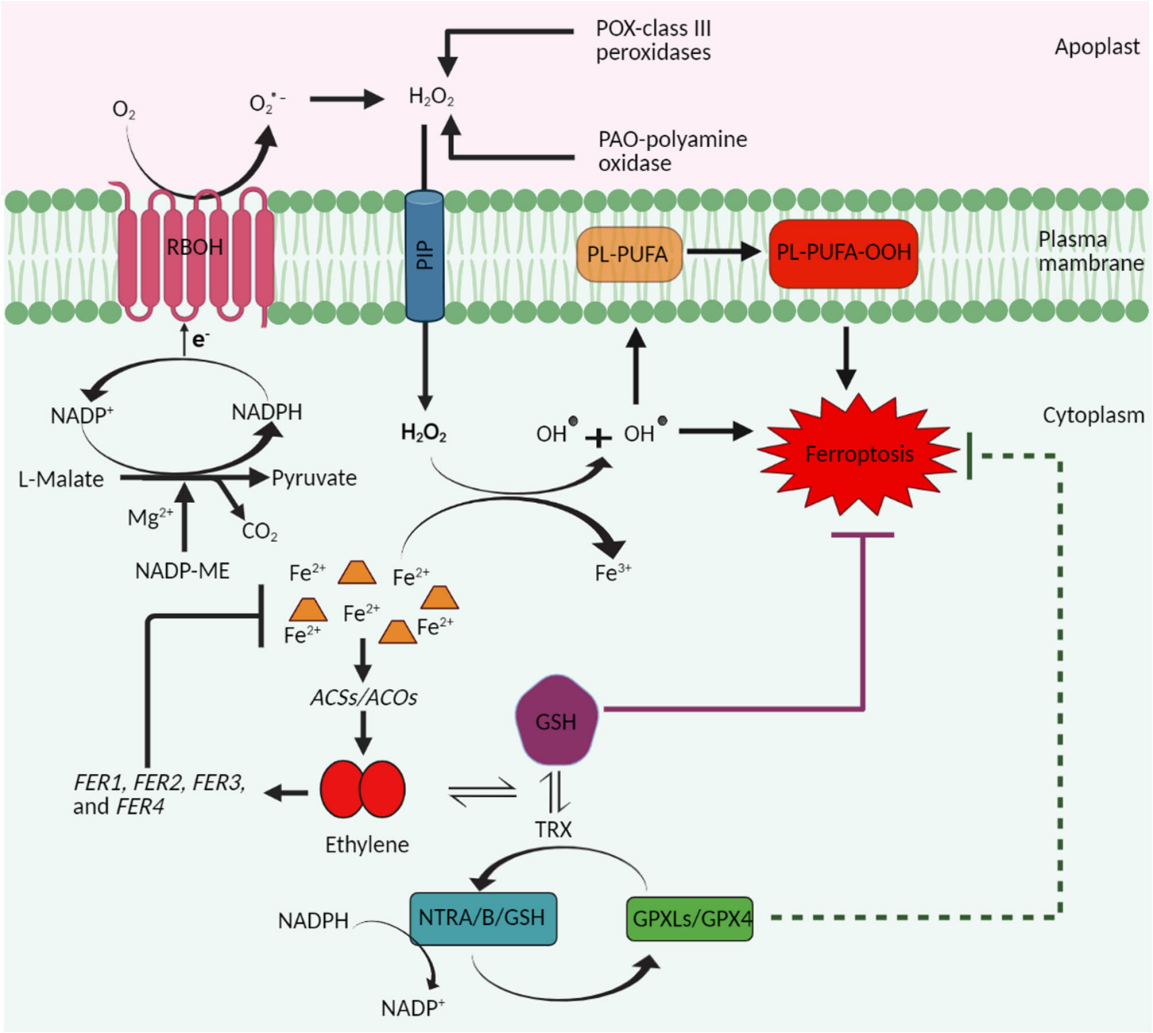
A schematic representation highlighting the proposed role of ethylene in ferroptosis cell death in plants. Plant ferroptosis is characterized by the increase in ROS and subsequent peroxidation of membrane lipids, accumulation of iron, and depletion of reduced glutathione (GSH). Stress-induced accumulation of ROS is mainly dependent on the activity of plasma membrane-localized NADPH-oxidase (respiratory burst oxidase homolog, RBOH) proteins. NADP-malic enzyme supplies electrons to the RBOH proteins and thus contributes indirectly to ROS production and thus to plant ferroptosis. H_2_O_2_ produced in the apoplast, because of the conversion of superoxide radicals (O_2_^•-^) and/or the activities of the class III peroxidases (POX) and polyamine oxidases (PAO), enters the cytosol through plasma membrane intrinsic proteins (PIP; aquaporins) and mediates the conversion of Fe^2+^ to Fe^3+^ through the Fenton reaction. Fe^2+^ are otherwise involved in the biosynthesis of ethylene by functioning as a cofactor of enzyme ACC oxidase (ACO). Ethylene so produced triggers the synthesis of ferritin proteins that participate in the sequestration of ions of iron and thus prevent their accumulation, which is a prerequisite for ferroptosis cell death. In addition, ethylene also induces the synthesis of GSH and thus helps to maintain the GSH levels to prevent ferroptosis cell death.

Based on the presented literature evidence, it can be speculated that ethylene controls and inhibits the ferroptosis process in plants by at least three ways: (1) by limiting the excess accumulation of Fe^2+^ in the cells via increasing the iron sequestrating ferritin proteins and thus inhibiting the generation of excess ROS based on Fe^2+^ accumulation, (2) by activating the antioxidant defense mechanism to limit the excess ROS accumulation in the cells, and (3) by facilitating the synthesis of GSH (Figure 1). However, further experimentations are required to investigate the ethylene biosynthesis and/or signaling during iron- and ROS-dependent ferroptosis in plants.

## Author Contributions

RR and RG conceived the idea. Both the authors were involved in the writing of the manuscript, contributed equally to the drafts, and gave final approval for publication.

## Conflict of Interest

The authors declare that the research was conducted in the absence of any commercial or financial relationships that could be construed as a potential conflict of interest.
