# Supplementary material for: Plausible Involvement of Ethylene in Plant Ferroptosis: Prospects and Leads
Source: Front Plant Sci. 2021 Jun 28;12:680709. doi: 10.3389/fpls.2021.680709 (PMC8273338; doi:10.3389/fpls.2021.680709)
Supplement: Supplementary file 1 [file Table_1.DOCX]

| **S. No.** | **Chemical** | **Role in ferroptosis** | **Mode of action** | **Reference** |
| --- | --- | --- | --- | --- |
| 1. | Acrolein | Inducer | Depletion of the GSH | Hajdinak et al, 2019 |
| 2. | Buthionine sulfoximine (BSO) | Inducer | Inactivation of GSH-dependent peroxidases | Yagoda et al., 2007; Dixon et al., 2012; Yang et al., 2014 |
| 3. | RSL3 | Inducer | GPX-4 inhibitor that leads to cell death | Hajdinák et al., 2019 |
| 4. | Erastin | Inducer | Inhibits GPX4 that led to accumulation of H_2_O_2_ | Dangol et al., 2019 |
| 5. | NADP-Malic enzymes (NADP-ME) | Inducer | Induce ROS and Fe^3+^ accumulation via providing electrons to the NADPH oxidases | Dangol et al., 2019 |
| 6. | Deferoxamine (DFO) | Inhibitor | Inhibits Fe^3+^ and ROS accumulation | Dangol et al., 2019 |
| 7. | Ferrostatin-1 (Fer-1) | Inhibitor | Inhibits Fe^3+^ and ROS accumulation and subsequent lipid peroxidation | Distéfano et al., 2017; Dangol et al., 2019; Hajdinák et al., 2019 |
| 8. | Diphenyleneiodonium (DPI) | Inhibitor | Inhibits H_2_O_2_ and Fe^3+^ accumulation and subsequent ferroptosis during incompatible interaction | Dangol et al., 2019 |
| 9. | Cytochalasin E (Cyt E) | Inhibitor | Inhibits the accumulation of Fe^3+^ and ROS through inactivation of the actin microfilament polymerization | Dangol et al., 2019 |
| 10. | Liproxstatin-1 | Inhibitor | To slow the accumulation of lipid hydroperoxides | Hajdinák et al., 2019 |
| 11. | Ciclopirox olamine (CPX) | Inhibitor | Intracellular iron chelator | Distéfano et al., 2017 |

**Supplementary Table 1:** Role of different chemicals as inducer or inhibitors of ferroptosis in plants.
